# An interactive sports video game as an intervention for rehabilitation of community-living patients with schizophrenia: A controlled, single-blind, crossover study

**DOI:** 10.1371/journal.pone.0187480

**Published:** 2017-11-13

**Authors:** Nobuko Shimizu, Tomohiro Umemura, Masahiro Matsunaga, Takayoshi Hirai

**Affiliations:** 1 Ishikawa Prefectural Nursing University, 1–1 Gakuendai, Kahoku, Ishikawa, Japan; 2 Aichi Medical University, 1–1 Yazakokarimata, Nagakute, Aichi, Japan; 3 Fukui Prefectural University, 4-1-1 Kenjojima, Matsuoka, Eiheiji-cho, Yoshida-gun, Fukui, Japan; University of Regensburg, GERMANY

## Abstract

Hypofrontality is a state of decreased cerebral blood flow in the prefrontal cortex during executive function performance; it is commonly observed in patients with schizophrenia. Cognitive dysfunction, as well as the psychological symptoms of schizophrenia, influences the ability of patients to reintegrate into society. The current study investigated the effects of an interactive sports video game (IVG; Nintendo Wii™ Sports Resort) on frontal lobe function of patients with schizophrenia. A sample of eight patients (6 male and 2 female; mean age = 46.7 years, standard deviation (SD) = 13.7) engaged in an IVG every week for 3 months in a controlled, single-blind, crossover study. Before and after the intervention we examined frontal lobe blood-flow volume using functional near-infrared spectroscopy (fNIRS), and assessed functional changes using the Frontal Assessment Battery, Health-Related Quality of Life scale, and behaviorally-assessed physical function tests. fNIRS revealed that prefrontal activity during IVG performance significantly increased in the IVG period compared with the control period. Furthermore, significant correlations between cerebral blood flow changes in different channels were observed during IVG performance. In addition, we observed intervention-related improvement in health-related quality of life following IVG. IVG intervention was associated with increased prefrontal cortex activation and improved health-related quality of life performance in patients with schizophrenia. Patients with chronic schizophrenia are characterized by withdrawal and a lack of social responsiveness or interest in others. Interventions using IVG may provide a useful low-cost rehabilitation method for such patients, without the need for specialized equipment.

## Introduction

Disorders in attention, concentration, memory, processing speed, and executive functions are among the many that strongly affect the social outcomes of schizophrenia. These various types of dysfunction have a substantial impact on the social prognosis of patients, along with positive symptoms such as visual hallucinations and delusions, and negative symptoms such as abulia and autosynnoia [1]. In recent years, progress has been made in hospital-discharge support for patients with mental illnesses who have undergone long-term hospitalization. However, effective methods of rehabilitation for chronic mental illness are severely lacking. Patients with mental illness commonly experience social exclusion and difficulty in their daily lives as a result of their condition, often leading to repeated hospitalization. A 2013 survey by the Ministry of Internal Affairs and Communications of Japan reported that many people with mental disorders have little to no contact with family or their communities and are effectively socially isolated [2]. Patients with mental illness who have difficulty in social functioning typically face difficulty managing specific daily tasks such as preparing meals, washing, cleaning, organizing, and bookkeeping, as well as social interactions such as using public institutions and maintaining relationships. Impaired executive function has a particularly strong impact on activities of daily living. Executive function is defined as the human ability to skillfully engage in purposeful, self-serving behavior [3]. Specifically, executive function includes planning and problem-solving abilities, such as making predictions, selecting goals, planning, organization, and beginning, executing and integrating goal-oriented behaviors. A framework defining such impairments was developed recently. As stated by WHO, “*The International Classification of Functioning*, *Disability and Health*: *ICF* was created with the objective of describing actual behavior.”[4] When we assess the daily functioning of individuals with schizophrenia using this ICF, difficulties in the application of learning and knowledge, general tasks and executing requests, communication, motor capacity, self-care, family life, interpersonal relationships, and daily life, as well as community, social, and civic life in major settings, which are characteristic of schizophrenia, become apparent. It is thought that these disease characteristics make social participation for individuals with schizophrenia difficult [1]. Accordingly, in order to promote their social participation, there is a growing need to switch from a symptom-oriented approach to a focus on the conceptualization of treatments which support the daily functioning of service users. Despite this, focus is currently being given to the aspects of the disorder related to mental functioning. It is necessary to focus on promising approaches which target the disorder from a wider point of view, including the spheres of daily functioning and participation. Frontal lobe activation underlies these functions, and healthy frontal lobe activity is thought to be a contributing factor in the prevention of social isolation in patients with mental disorders [3]. Moreover, there is evidence that patients with chronic schizophrenia commonly exhibit hypofrontality, which is characterized by reduced blood flow into the frontal lobes relative to other areas of the brain at rest. Consequently, hypofrontality has received significant attention in schizophrenia research [5]. However, there are few effective methods for improving cognitive function in individuals with schizophrenia. In addition, there have been few studies of interventions for improving motor function [6].

Meanwhile, there have been many reports of the development of programs targeting older individuals who tend to engage in limited habitual exercise [7–11]. Dual-task training has been reported to be an effective intervention, not only for motor function, but also cognitive function [7,12]. This training includes physical movement to match the rhythm of music (eurhythmics) and the performance of simple math calculations while walking. Bridenbaugh and Kressig [7] proposed that neural circuits are first developed as a foundation for music learning by reproducing musical gestures and physical movements, and that repeated perception of movement elements of music through muscular movement stimulates these neural circuits. Thus, there is evidence that dual-task training stimulates motor and cognitive functions simultaneously, thereby improving functioning in both domains.

However, although it is possible for patients with schizophrenia to participate in physical-exercise programs while hospitalized or attending psychiatric-care centers, it is difficult for such individuals to voluntarily participate in group-exercise programs after returning to their communities following discharge [1]. As a result, most patients with schizophrenia stop exercising after discharge.

Recent developments in engineering have enabled the development of interactive video game environments that can be presented via home game consoles. A number of studies in the last 2–3 years have tested computer games as potential therapeutic intervention methods [13–16]. One study reported an improvement in depression symptoms and cognitive function in elderly people with mild depression following 3 months of regular 35-minute sessions of Nintendo’s Wii™ sports games that included an exercise program [13]. In addition, Kühn and colleagues [14] revealed that cerebral areas involved in memory construction, spatial orientation, strategic planning, and fine motor skills all improved in subjects who played computer games for 30 minutes per day for 2 weeks, compared with a control group. Another study examined the effects of an intervention using a computer-based cognitive-training program (CogniPlus) that targeted improvement of cognitive functions in patients with Parkinson’s disease, compared with patients who engaged in a motion-control computer game (Nintendo Wii™) [15]. The results revealed that the Wii training group exhibited better attentional function than the CogniPlus group [15].

These findings indicate the potential value of computer-based therapeutic interventions for patients with mental illnesses such as schizophrenia or Alzheimer’s disease, which are known to cause atrophy in particular parts of the brain. However, there have been no studies of interventions that specifically targeted patients with schizophrenia.

Deutsch et al. reported that interactive sports video games could increase the effects of rehabilitation, as they are highly versatile and increase patient motivation [16]. Interactive, near virtual-reality video environments presented as computer games can incentivize patients to exercise, facilitating exercise interventions while maintaining interest. Thus, interactive sports video games may allow patients with schizophrenia who feel uncomfortable exercising to engage in physical activity more comfortably than conventional exercise interventions.

Interactive sports video games provide a unique medium suited to the achievement of several requirements for effective interventional rehabilitation. Specifically, therapy can be provided within a functional, purposeful and motivating context.

In the present study, we investigated the therapeutic value of an intervention using interactive sports video games presented via a home console. This intervention method required simultaneous activation of motor and cognitive functions, which have been shown to be highly versatile and encourage patient compliance, and which can influence the frontal lobes of patients with schizophrenia who exhibit mild decline of frontal lobe function [14,15]. In the current study, we used near-infrared spectroscopy (NIRS) imaging to visualize frontal lobe activation. NIRS probes can be easily fixed to a subject’s head, allowing for recording in diverse experimental conditions. NIRS does not restrict subjects’ movements, thus enabling measurement in natural postures and while moving. This functional brain-imaging method is unique in its measurement of changes in hemoglobin concentration in cerebral structures to evaluate blood flow and oxygen metabolism. Similarly to functional magnetic resonance imaging (fMRI), NIRS enables the evaluation of cerebral activation during working-memory tasks [16, 17].

Interactive sports video games (IVG) can easily be used independently in the home. The current study tested the effects of an IVG exercise program on physical and cognitive function as well as health-related quality of life (HRQOL) in patients with schizophrenia, with a practice period of 3 months. This method was examined as a potential intervention for increasing daily motivation to improve and maintain cognitive function in patients with schizophrenia living in the community, thus potentially aiding their reintegration into society.

## Materials and methods

### Subjects

**Fig 1:**
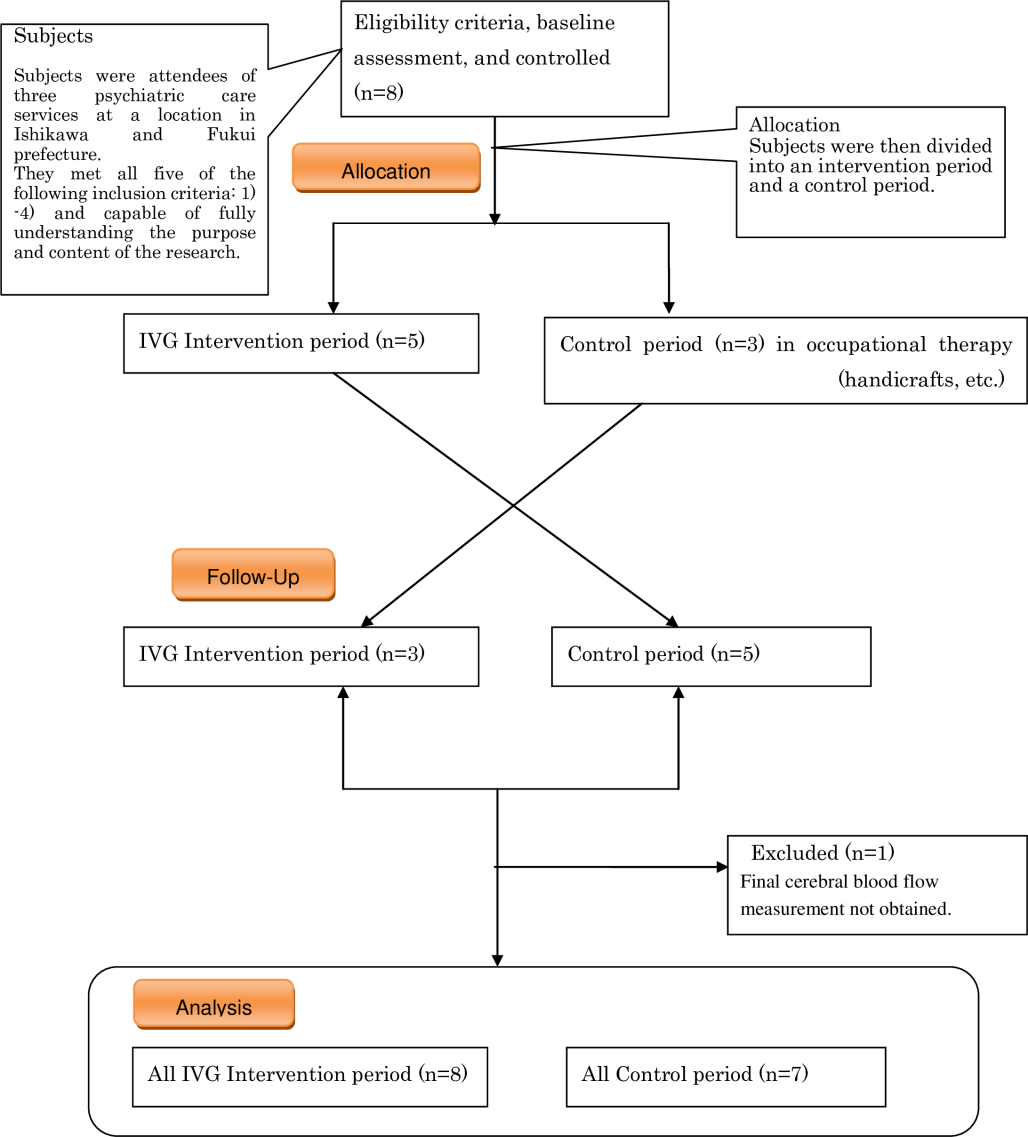
Flowchart showing enrollment and data analysis of study subjects.

Subjects were attendees of psychiatric care services at one location in Ishikawa prefecture and two locations in Fukui prefecture (Fig 1). They met all five of the following inclusion criteria: 1) diagnosed with schizophrenia according to the criteria defined by the International Classification of Diseases (the 10th revision of the International Statistical Classification of Diseases and Related Health Problems; ICD-10); 2) not diagnosed with a mental disability, alcoholism, or drug addiction; 3) aged 18–64 years; 4) scored 40 points or more on the Global Assessment of Functioning (GAF) test, indicating a non-severe mental state; and 5) capable of fully understanding the purpose and content of the research. The nature and purpose of the study was explained to all subjects, and written consent to participate in the study was obtained. Each patient’s principal physician’s permission was also obtained. We performed the power analysis in G Power version 3.1.9.2 [18]. The results indicated that within the group, power was acceptable when n = 19 with the Wilcoxon signed-rank test (matched pairs; two-tailed t-tests; effect size = 0.9; alpha error = 0.05; 1—beta error = 0.95); between groups required n = 70 with the Mann—Whitney U test (two groups; two-tailed t-tests; effect size = 0.9; alpha error = 0.05; 1—beta error = 0.95).

**Fig 2:**
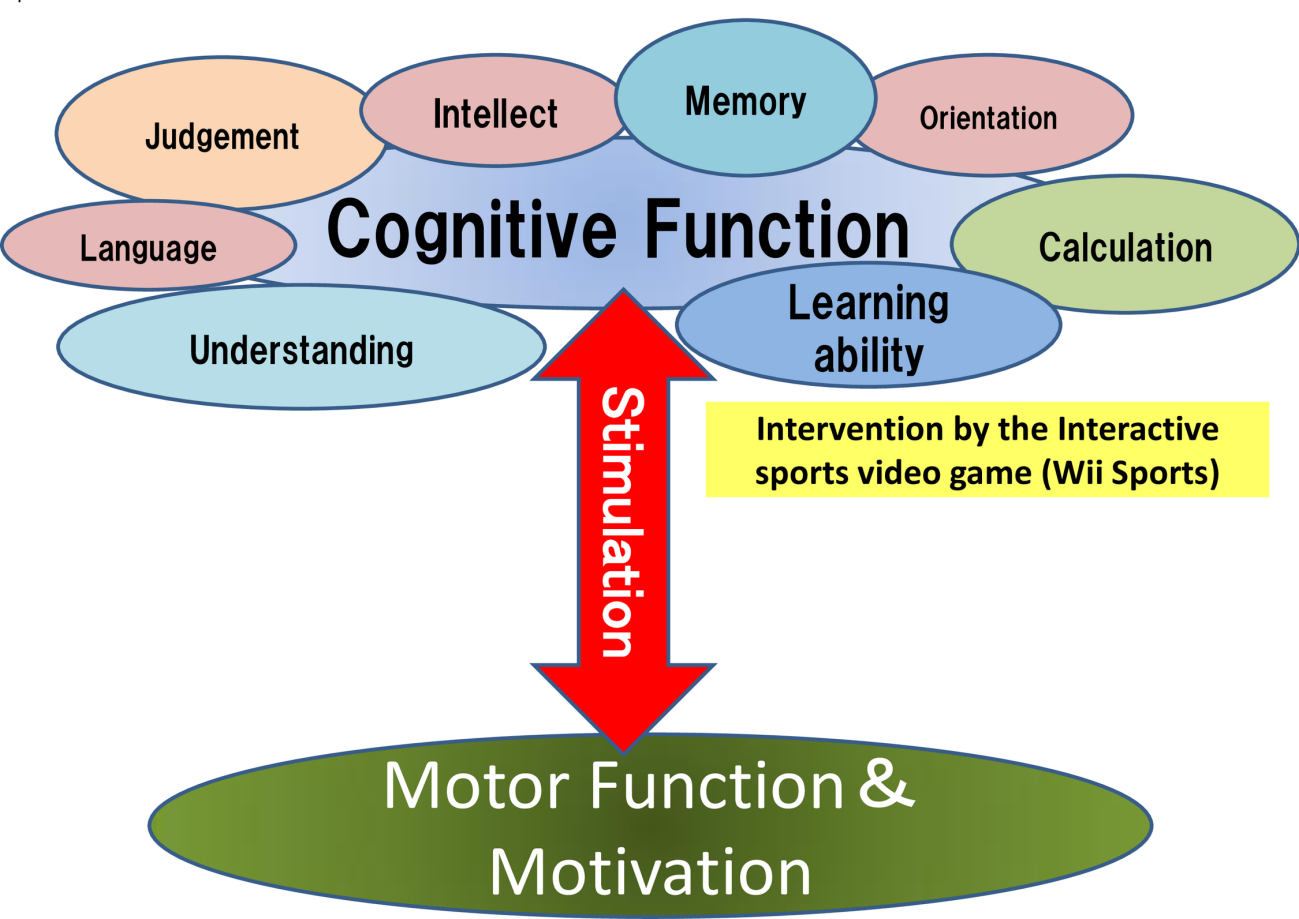
Interactive sports video game (IVG) intervention.

**Fig 3:**
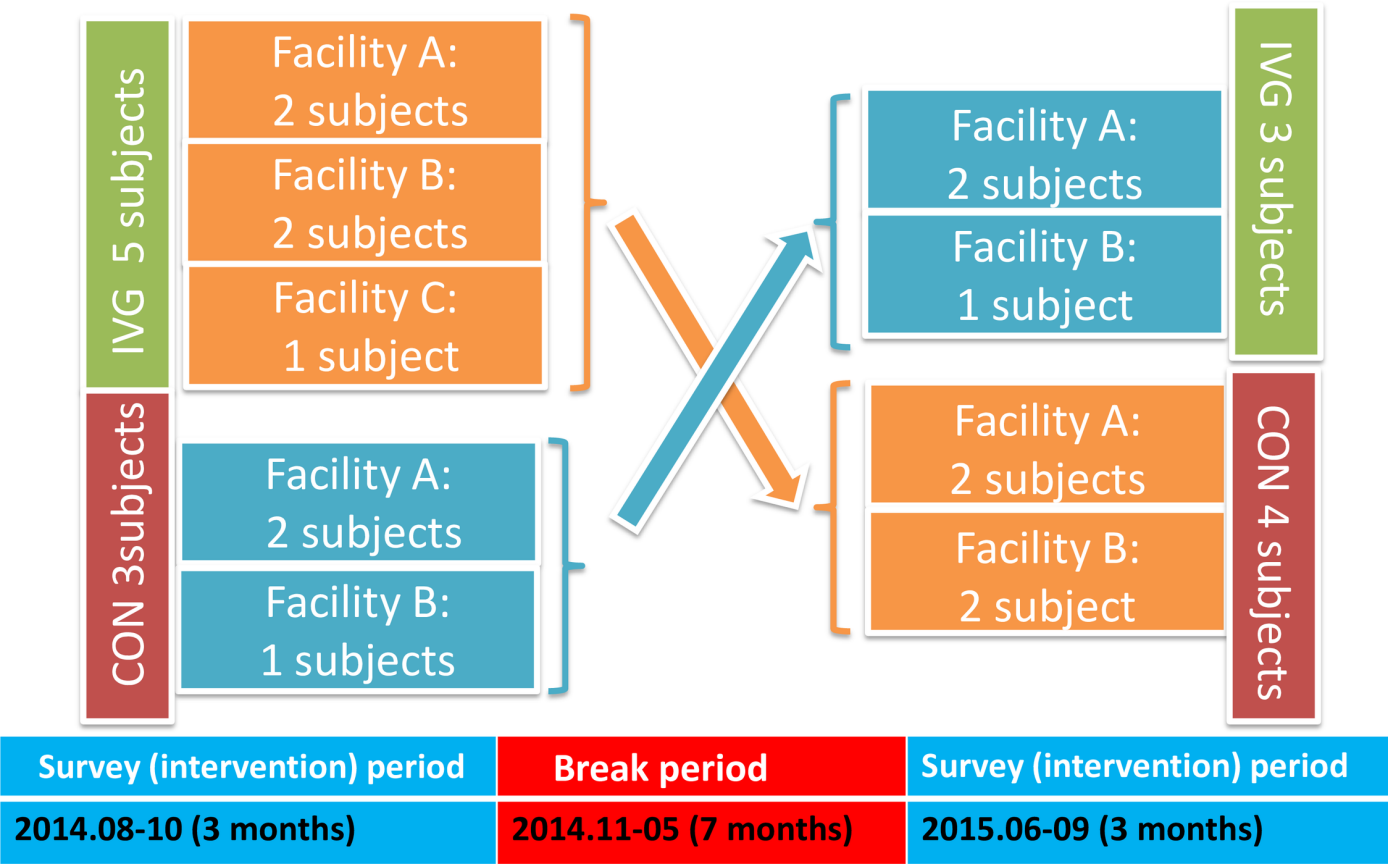
Intervention and survey periods. Crossover experimental design: IVG period (8 subjects), CON period (7 subjects).

**Fig 4:**
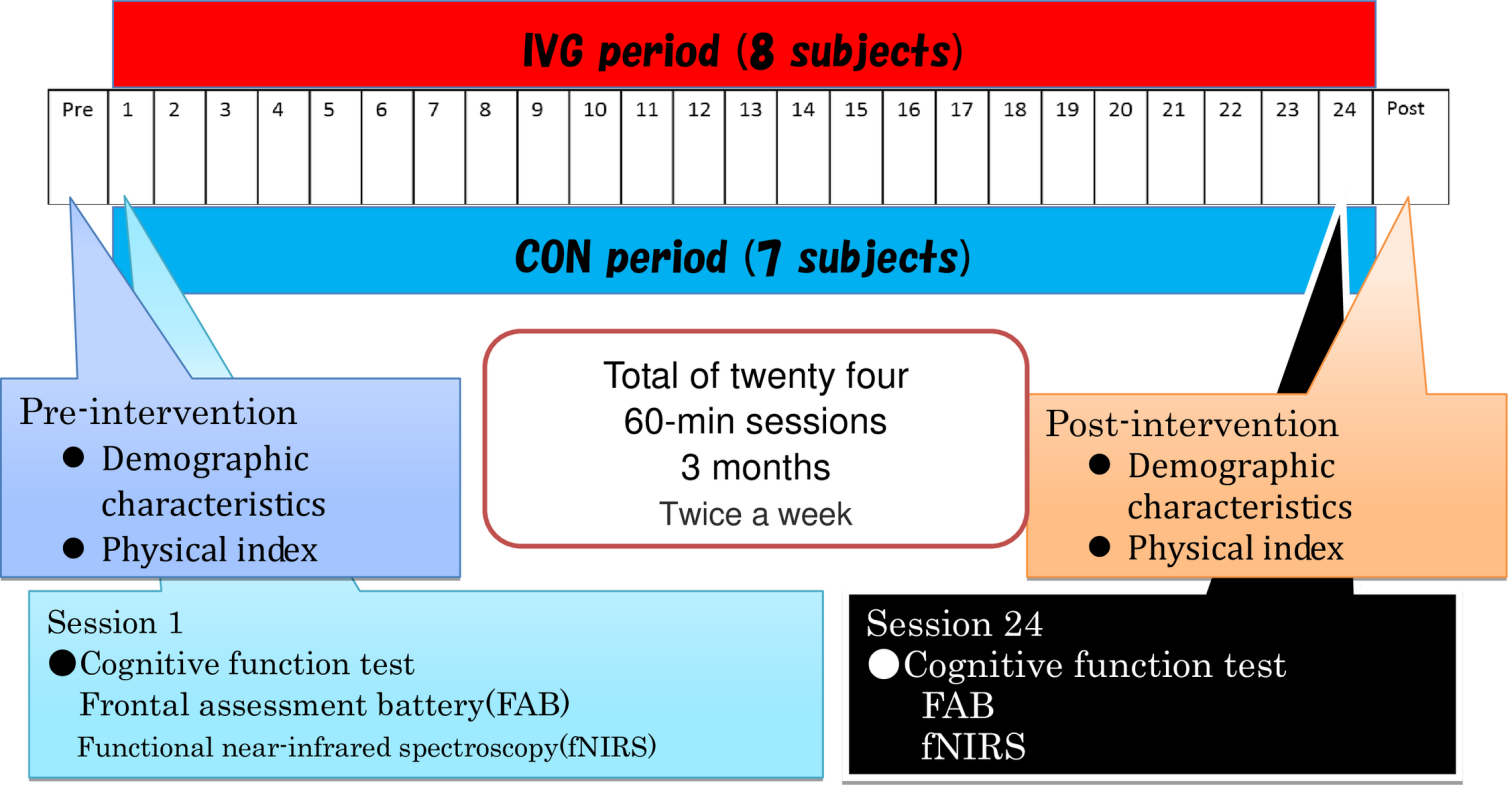
Research category and measurement schedules.

The subjects participated in occupational therapy (handicrafts, etc.) at their psychiatric center during the control period (Fig 1). The survey and measurement period took place over approximately 2 years (25 months), including 3 months starting August 2014, a break period of 7 months, and another 3 months starting June 2015 ((Fig 2, Figs 3 and 4). Measurement schedules took place in the first and last weeks (Fig 4).

### Ethical considerations

Before starting the survey, we explained the nature and purpose of this study as well as any risks associated with it. The survey and research were conducted after subjects had provided informed consent. This study was approved by the Ethics Committee of the Ishikawa Prefecture Nursing University (Kandai no. 337). Clinical trial registration details are as follows: Clinical Trial Registry Number UMIN000023766 2016/08/25 [University Hospital Medical Information Network (UMIN) Center], retrospectively registered.

### Intervention method

The intervention used a Nintendo Wii™ console software program called “Wii™ Sports Resort” that includes 12 sports (Table 1). Performance requires fine movements with the Wii remote control, resulting in motions similar to those involved in performing the actual sport. To perform the task, the subject was required to stand in front of the screen and use his or her whole body to manipulate the remote control, while playing against a computerized opponent on the monitor in each game. This intervention was implemented as one 60-minute session twice a week for 3 months. In the control period, the same subject participated in a normal occupational therapy program over the same period 1 year later (Jun–Sep), and measurements were taken in the 3 months before and 3 months after participation. Thus, the subject that initially underwent the Wii intervention participated in the activities of the control period, and the subject that was in the control period to start with participated in the Wii intervention during the same period in the following year (Fig 3).

**Table 1:** The twelve sports included in Wii™ sports resort.

Easy sports	Ball sports	Leisure sports
Chanbara	Basketball	Canoeing
Biking	Ping pong	Wakeboarding
Frisbee	Golf	Sky Ranger
Archery	Bowling	Marine Biking

### Questionnaires and demographic data

#### Basic characteristics

Nurses interviewed subjects and took measurements to determine each subjects’ family situation, chief complaint, medical history, drug therapy, family history, height, weight, and blood pressure.

#### Measurement of general mental function

We verified that the GAF score was 40 points or above based on diagnostic information from the principal physician of each subject.

#### Frontal assessment battery (FAB) [19]

The FAB was developed by Dubois et al. [19,20] (http://dementiakt.com.au/wp-content/uploads/2016/03/11_FAB.pdf FAB English version; http://yoshiya-hasegawa.com/life_doctor/fab.pdf FAB Japanese version). It consists of six tasks believed to involve the frontal area of the frontal lobe. Tests consist of similarities (conceptualization), lexical fluency (mental flexibility), motor series (motor programming), conflicting instructions for studying sensitivity to interfering stimuli and two-one tapping tasks (sensitivity to interference), go/no-go tasks (inhibitory control), and prehension behavior (environmental autonomy). The maximum score is 18 points. This assessment is used widely in clinical situations, as it can be conducted easily in less than 15 minutes, and the confined range of results allows for intuitive evaluation of the patient.

#### HRQOL scale (SF-36) [21,22]

The SF-36v2® scale is the standard scale used for measuring HRQOL. We used the revised version of the original SF-36 (Japanese translation version 1.2. https://www.sf-36.jp/qol/files/sf-36v2a.pdf SF-36 Japanese version; http://www.brandeis.edu/roybal/docs/SF-36_website_PDF.pdf SF-36 English version) The SF-36 is composed of multiple questions that evaluate the following eight health concepts: (1) physical function (PF), (2) daily role functioning (physical; RP), (3) physical pain (BP), (4) perceptions of general health (GH), (5) vitality (VT), (6) functions in social activities (SF), (7) daily role functioning (psychological; RE), and (8) general mental health (MH) [21, 22].

### Measurement of behaviorally-assessed physical functioning

We behaviorally (as opposed to self-reportedly) assessed physical function using the following six items: (1) skillfulness and agility (timed up and go), (2) flexibility (functional reach test), (3) balance (one-leg standing test with eyes open), (4) gait (walk 10 m at maximum speed, (5) muscle endurance (30-second chair test), and (6) muscle force (grip strength).

#### Skillfulness and agility (timed up and go) [23]

For the timed up and go test, we measured the time taken for subjects to stand up from a chair, walk 3 m, turn around, walk back to the chair and sit down again. Subjects were instructed to walk in a straight line at a comfortable and safe pace.

#### Flexibility: Functional reach test [24]

In this task, the subject stood with both legs widely apart with the side of his or her body lined up against a wall. The subject was instructed to make loose fists with his or her hands, and to raise his or her arms so that they were perpendicular to the body. The ends of his or her fists, which were raised to shoulder height, were marked and the arm farthest from the wall was then put back down. While maintaining the same height with the other fist, the subject was asked to extend his or her hand forward as far as possible without moving his or her feet. The experimenter marked the point at which the subject’s arm was extended furthest and then instructed the subject to return slowly to the original position. The measurement was then repeated. The distance between the original position and the maximally extended position was measured, and the results were recorded to one decimal place.

#### Balance: One-leg standing test with eyes open

In the one-leg standing test with eyes open [25], subjects were instructed to stand with their hands on their waist, raise an arbitrarily chosen foot approximately 20 cm from the floor, and hold their balance. We recorded the time taken for subjects to lose their balance (maximum: 120 s). Loss of balance was defined as shifting the sole of the non-lifted foot from its original position, removing the hands from the waist, or touching the floor with any body part other than the pivot leg. The test was repeated twice, with the higher value recorded for analysis.

#### Gait: Maximum walking speed (10 m)

Maximum walking speed was defined as the time taken to walk 10 m in a straight line after being instructed to walk as quickly as possible. The time taken was recorded with a walking-measurement instrument (T.K.K.5801; Takei Scientific Instruments Co., Ltd.) equipped with a stopwatch function. The shorter time of two trials was recorded for analysis.

#### Muscle endurance (30-s chair test)

The 30-s chair test (CS-30) [26] is a safe and effective test for older adults, which is used to assess muscle strength in the lower extremities. The test began with subjects seated on a chair with their back straight, feet flat on the floor approximately shoulder-width apart, and arms crossed against the chest. At the “go” signal, subjects rose to a full standing position (with the body erect and straight) before returning to the original sitting position. Subjects were instructed to complete as many full stands as possible within 30 s. The score was the total number of stands executed.

#### Muscle force: Grip strength

To assess grip strength in both hands, subjects held digital handgrip dynamometers (T.K.K.5401; Takei Scientific Instruments Co., Ltd.) in each hand at the sides of the body and were instructed to squeeze the devices as hard as they could during expiration. They were instructed not to move their hands or touch the dynamometer to their body. The values for each hand were analyzed.

### Cerebral blood flow measurement

We measured subjects’ cerebral blood flow while playing the interactive sports video games using functional near-infrared spectroscopy (fNIRS). In the fNIRS paradigm, infrared light with a wavelength of approximately 800 nm is emitted from above the scalp, and used to measure cerebral blood flow by capturing changes in hemoglobin concentration that occur with neural activity in the brain. The Shimadzu LABNIRS (Shimadzu Co. Ltd., Kyoto, Japan) used in the current study was developed for research purposes, and measures three wavelengths (780 nm, 805 nm, and 830 nm) to detect changes in light absorption, allowing calculation of changes in concentrations of oxygenated and deoxygenated hemoglobin (oxyHb and deoxyHb). We focused on increases in oxyHb, because this appears to reflect task-related cortical activation more directly than decreases in deoxyHb. We defined the patterns of NIRS waveforms using the integral value of the grand-average waveforms in the frontal region. The integral value describes the size of the hemodynamic response during the 60-second activation task period [27,28].

#### Task

Originally, as an integrated part of the occupational therapy program, participants had the experience of playing Wii for about ten minutes once every two to three months under the guidance of an occupational therapist. Participants could freely choose their preferred game from the twelve choices, and thus developed preferences for different games. In our task, all participants chose their preferred two or three games and played them for the 60 minutes. The reason participants were not limited strictly to the same game is that, due to the disease characteristics, it was difficult for participants to keep playing only a game designated by the coach, and it was anticipated that motivation would decrease and it would be difficult to maintain the intervention.

Consideration was also given to preventing participants from choosing by themselves to skip day care, because the non-intervention (control) group continued their usual day care programs, other than no longer being allowed to exercise or play Wii, per their normal routines; they were in fact allowed to participate freely in activities they chose (excluding the “Chanbara” (Iaigiri) game used in the measurement task).

Of the 12 games in Wii™ Sports Resort, we selected the “sword fighting” game “Chanbara” (*Iaigiri*). In this game, subjects are required to slash objects that are approaching them on the screen. Responses are required within 2–3 s, and subjects are instructed to respond faster than their opponent. Objects included furniture, fruits, and clocks. This game requires players to make rapid judgments and responses.

#### Measurement site: Bilateral measurement of frontal lobe activation

**Fig 5:**
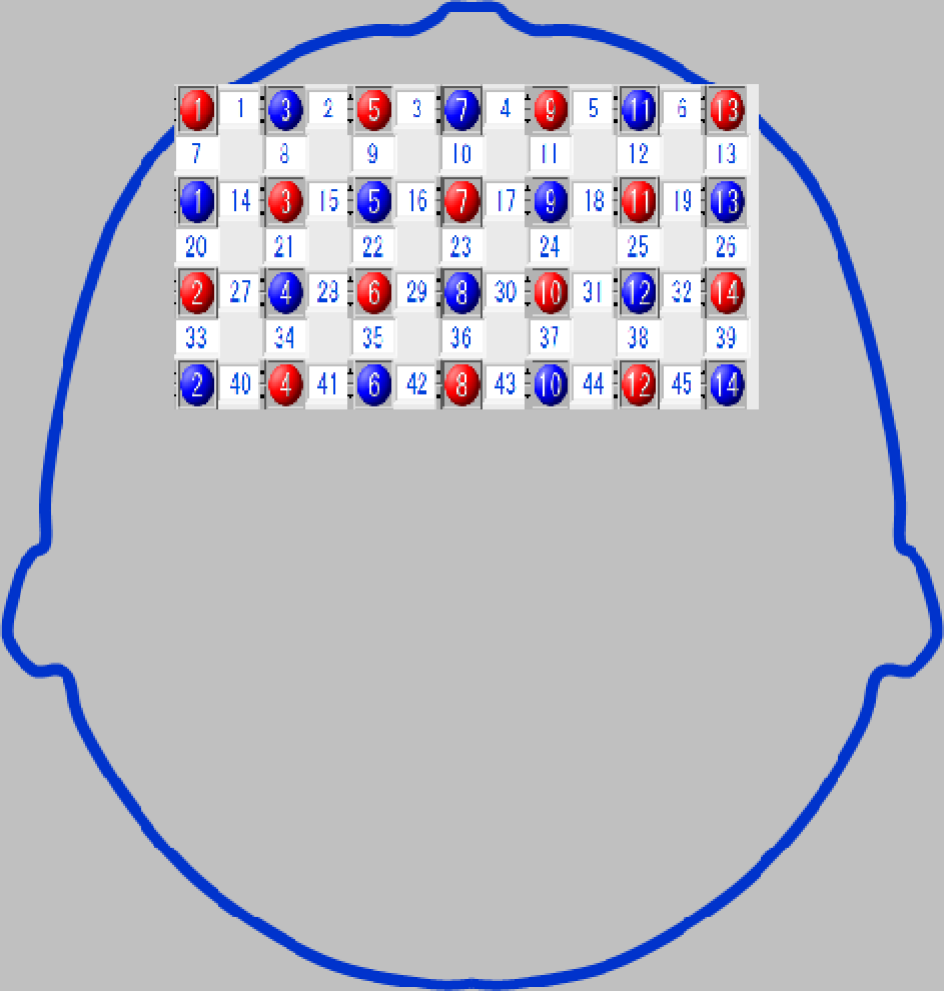
Regions measured with near-infrared spectroscopy (NIRS). Identification of the attachment region was conducted in accordance with the International 10–20 system, with the probe holder set so that the light-transmitting probe 7 (T7) was directed towards the frontal pole of the frontal processing zone.

**Fig 6:**
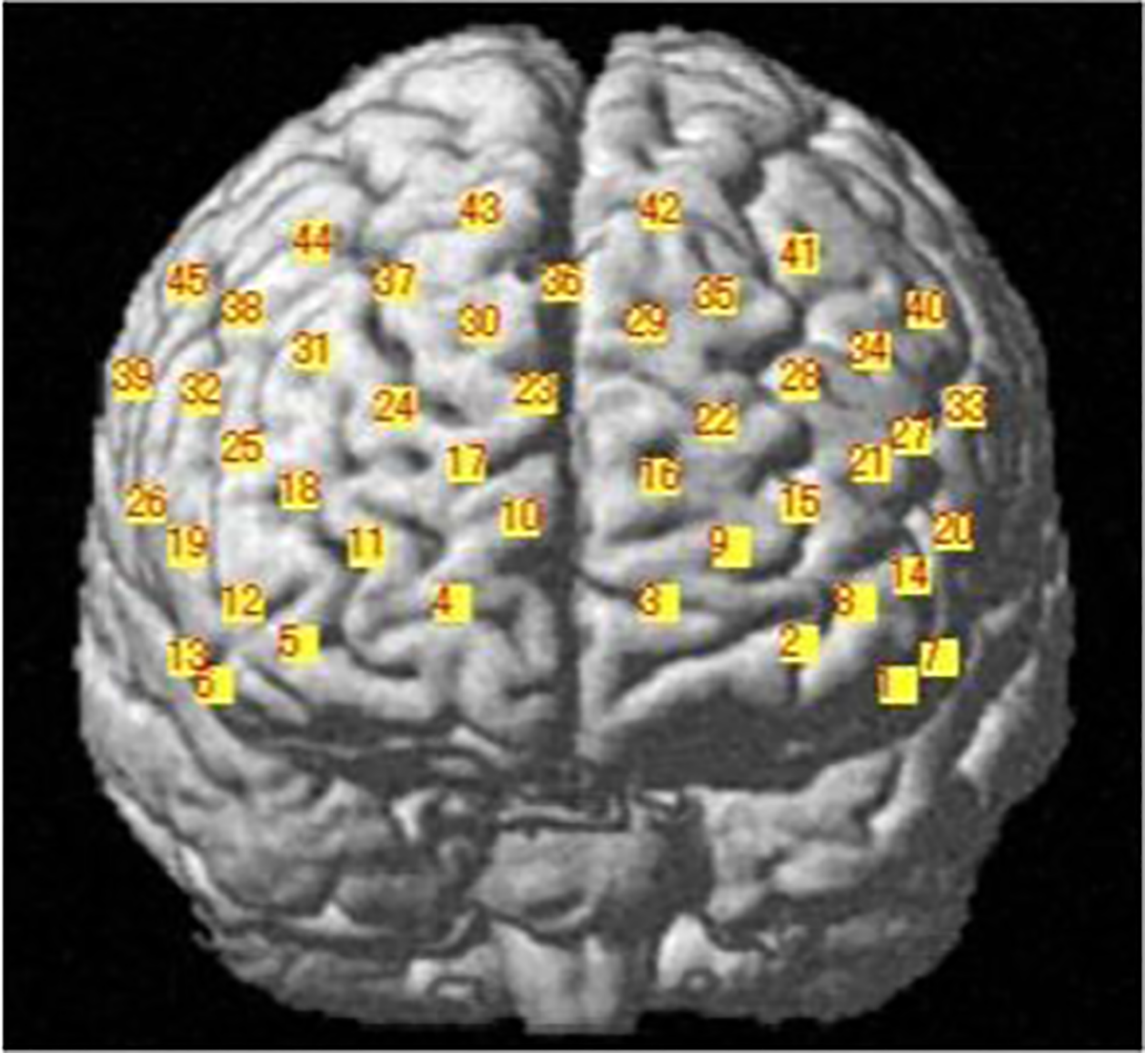
Specified brain coordinate locations using statistical parametric mapping for near-infrared spectroscopy (NIRS-SPM).

**Fig 7:**
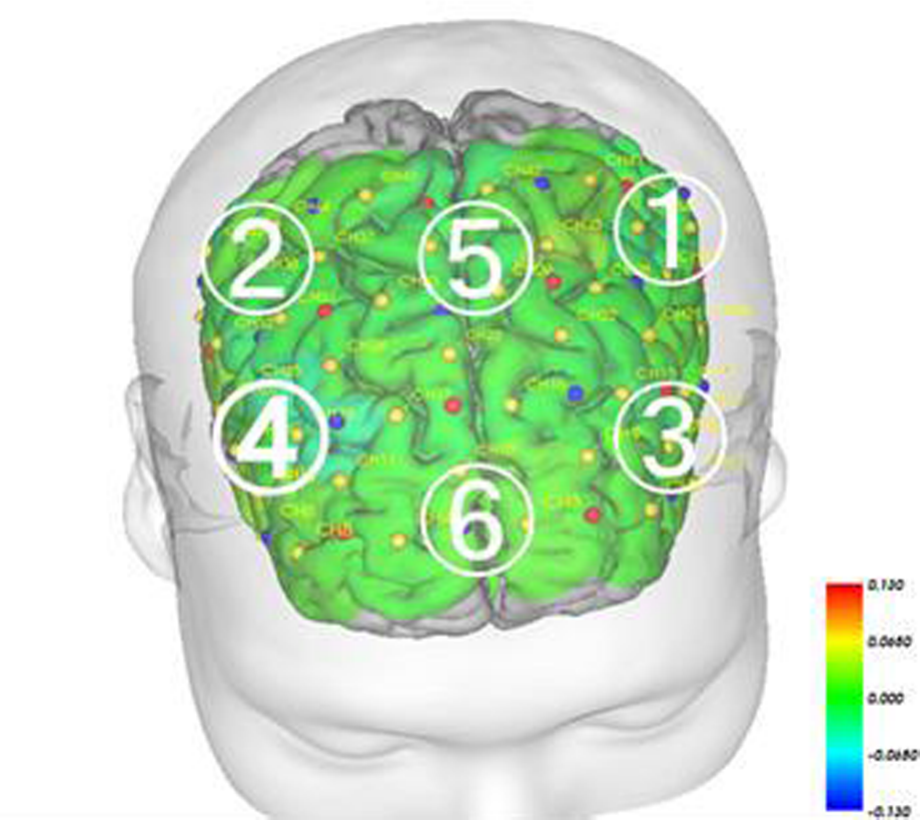
Brodmann area 10. BA10 is in the anterior frontal lobe. 1, Left dorsolateral prefrontal cortex; 2, Right dorsolateral prefrontal cortex; 3, Left ventrolateral prefrontal cortex; 4, Right ventrolateral prefrontal cortex; 5, Medial prefrontal cortex; 6, Orbitofrontal frontal cortex.

We used probabilistic registration, which can register channel position on MNI standard brain coordinates based on the international 10–20 method, even when MRI images are not available [29]. We used the flexible holder produced by Shimadzu Co. Ltd., using a 3D digitizer to position the probe holder so that the light-delivery probe 7 (T7) would be positioned on front polar zero (Fig 5). We then changed the channel positions to the standard brain-coordinates using statistical parametric mapping for near-infrared spectroscopy (NIRS-SPM) and projected them to the cortical surface (Figs 6 and 7).

#### Time protocol

**Fig 8:**
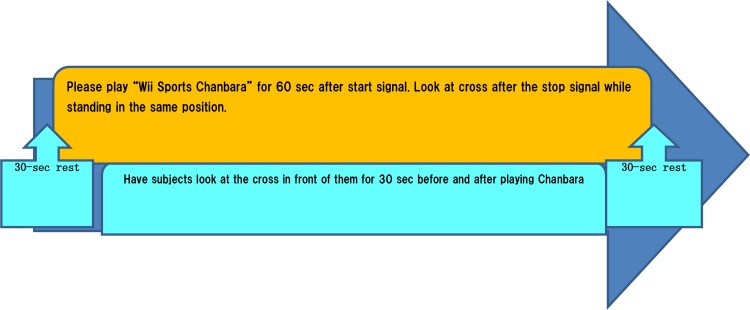
Tasks for cerebral blood flow measurement.

Each 120-s trial consisted of 30 s rest + 60 s task + 30 s rest (Fig 8). Subjects took a 30-s rest before and after executing the task. In the rest period, subjects were instructed to stare at a cross in front of their eyes without thinking about anything specific. The instructor signaled the beginning and end of each resting period.

### Analysis

We used IBM SPSS 21.0J for Windows (Tokyo, Japan) for all statistical analyses, with a significance level of p < 0.05.

#### Changes before and after intervention

We compared the behaviorally-assessed physical functioning measurements, SF-36, and FAB scores between the intervention period and the control period. Because our data did not fit a normal distribution, baseline comparative evaluation was done with nonparametric Mann-Whitney U tests. Next, the values before and after the intervention within the group were evaluated by the Wilcoxon signed-rank test.

#### Evaluation of cerebral blood flow measurement

To evaluate task-related changes, we analyzed the differences between the integral value of oxyHb before and after task performance in the intervention and control periods, identifying regions of interest through our brain coordinate information (Figs 5 and 6). Baseline comparative evaluations were performed via nonparametric Mann-Whitney U tests; the values before and after the intervention within the group were evaluated by Wilcoxon signed-rank tests. The differences in the integral oxyHb values before and after task performance in the intervention and control periods were assessed using the nonparametric Kruskal-Wallis test. We used the Kruskal-Wallis test to analyze cerebral blood flow. Being unsure of the distribution it followed, we chose nonparametric analysis. Values before and after the intervention were assigned to groups 1 and 2, and values before and after the control group were assigned to groups 3 and 4; rates of cerebral blood flow measured by a plurality of the channels at a given time were used as the test variables.

To assess the network of intervention-related activated brain regions we examined Spearman’s correlations between the changes in integral oxyHb before and after the intervention, for both the intervention and control periods for each brain region (channel 45). Based on these correlations, relationships (i.e., networks) between the brain sites activated during the task in the two periods were graphed and compared.

## Results

### Subject characteristics

Of the 12 subjects who consented to take part in the study, eight (mean age: 46.7 standard deviation (SD): 13.7 years) completed both the pre- and post-intervention assessments, and were included in the final data analysis. The characteristics of the 12 subjects are shown in Table 2, which also shows the data of the eight participants in the intervention period (IVG period) and seven in the control period (CON period).

**Table 2:** Participant characteristics.

Subjects	Sex	n
All subjects	Male	8
	Female	4
Analyzed subjects	Male	6
	Female	2
Age range (years)(mean and SD age)	22–64	
	(46.7 ± 13.7)	

### Behaviorally-assessed physical functioning before and after intervention

The results of the comparison of the behavioral assessments of physical function before and after intervention are shown by test item in Table 3 (i.e., muscle force [grip strength], balance [one-leg standing test with eyes open], flexibility [functional reach test], skillfulness/agility [timed up and go], gait [10 m walk at maximum speed], leg strength/endurance [30-s chair test]). The paired t-tests revealed a significant difference between balance performance in the intervention period and muscle force in the control period. However, no significant differences were observed in the Mann-Whitney U-tests when comparing the intervention and control periods.

**Table 3:** Pre- and post-intervention comparison of behaviorally-assessed physical functioning test performance and FAB scores in each period.

		Pre-intervention at baseline			Post-intervention			Difference pre- and post-intervention		
		IVG period (n = 8)	CON period (n = 7)	Mann- Whitney U test (P value)	IVG period (n = 8)	CON period (n = 7)	Mann- Whitney U test (P value)	IVG period Wilcoxon signed-rank test (P value)	CON period Wilcoxon signed-rank test (P value)	Mann- Whitney U test (P value)
Body balance (one-leg standing eyes open)	s	21.67 (8.70–48.30)	19.80 (7.33–72.00)	0.297	30.09 (12.80–120.00)	9.35 (4.83–120.00)	0.115	0.297	0.115	0.121
Flexibility (sit-and-reach test)	cm	37.00 (33.25–41.50)	34.00 (31.75–39.00)	0.256	34.50 (31.00–38.25)	38.00 (24.00–40.00)	0.696	0.256	0.696	0.298
Gait (10 m walking speed)	s	3.23 (2.95–3.68)	4.36 (3.54–5.62)	0.262	4.10 (3.28–4.65)	5.33 (3.56–6.12)	0.302	0.262	0.302	0.606
Muscle endurance (CS-30)	times	20.50 (11.75–27.00)	17.00 (12.00–21.00)	0.148	21.00 (12.00–28.75)	18.00 (13.00–18.00)	0.068	0.148	0.068	0.590
Muscle strength (right grip strength)	kg	34.75 (27.38–41.25)	29.50 (20.38–38.63)	0.149	37.75 (33.38–39.63)	41.50 (25.00–44.00)	0.905	0.149	0.795	0.261
Functional mobility (timed Up & Go test)	s	4.12 (3.18–4.44)	4.70 (4.1–6.8)	0.092	4.29 (3.71–4.61)	4.42 (3.97–6.52)	0.606	0.092	0.606	0.302
FAB score		15.50 (9.75–18.00)	15.50 (12.50–17.00)	0.932	16.00 (12.00–18.00)	15.00 (14.00–18.00)	0.916	0.534	0.078	0.835

Notes: Data are presented as the median (interquartile range). CON, control; IVG, interactive sports video game https://figshare.com/s/7b1e2336743df3e76b06

### HRQOL before and after intervention

Comparison of changes in HRQOL (SF-36) measurements before and after intervention in the intervention and control periods revealed significant improvement in three of the eight items: bodily pain (BP), social functioning (SF), and role/emotional score (RE) (Table 4).

**Table 4:** Pre- and post-intervention differences for the 8 subtests of the SF-36v2™ in each period.

	Pre- intervention at baseline			Post- intervention			Difference pre-and post -intervention			
	IVG period (n = 8)	CON period(n = 7)	Mann-Whitney -U (P-value)	IVG period (n = 8)	CON period(n = 7)	Mann-Whitney -U (P-value)	IVG period Wilcoxon signed-rank test(P-value)	CON periodWilcoxon signed-rank test(P-value)	Mann-Whitney -U (P-value)	Effect size (r)
PF (physical functioning)	46.33(27.85–55.13)	51.61(29.61–55.13)	0.795	51.61(37.53–58.66)	55.13(44.57–58.66)	0.814	0.027	1.000	0.243	0.30
PR (physical role functioning)	37.49(32.37–54.54)	37.49(30.67–51.98)	0.931	46.01(28.96–52.83)	46.01(42.69–52.83)	0.643	0.399	0.593	0.195	0.34
BP (bodily pain)	48.59(34.65–59.65)	52.13(38.96–59.65)	0.549	49.03(45.49–61.42)	61.42(52.57–61.42)	0.272	0.138	0.593	0.041	0.47
GH (general health perceptions)	46.19(39.97–50.92)	53.76(48.89–63.49)	0.109	46.19(42.41–54.30)	48.89(46.19–54.30)	0565	0.248	0.593	0.437	0.20
VT (vitality)	50.24(41.79–53.32)	54.86(51.01–65.62)	0.154	53.32(47.17–65.62)	56.40(56.40–62.55)	0.419	0.115	0.655	0.692	0.10
SF (social role functioning)	43.94(39.91–53.80)	57.10(27.50–57.10)	0.369	43.94(43.94–50.52)	57.10(57.10–57.10)	0.031	0.450	1.000	0.025	0.58
RE (emotional role functioning)	41.69(36.37–55.50)	50.19(27.75–55.50)	0.797	52.31(43.81–56.56)	43.81(35.31–52.31)	0.295	0.072	0.285	0.034	0.55
MH (mental health)	45.13(41.14–51.11)	54.43(45.79–63.07)	0.088	43.80(43.80–54.43)	54.43(49.11–54.43)	0.232	0.340	0.414	0.241	0.30

Notes: Data are presented as the median (interquartile range). CON, control; IVG, interactive sports video game https://figshare.com/s/251ab69e9c548bfa4667

### Changes in frontal assessment battery (FAB) scores and activation in the bilateral frontal lobe

The Mann-Whitney U-test did not reveal any significant changes in FAB scores before and after intervention (Table 3). We measured blood-flow changes during task performance, before and after intervention in the intervention and control periods. In addition, comparison of intervention-related changes revealed significantly more activation in channels 27 (P = 0.021, r = 0.60 (Effect size, medium-to-large) and 41 (P = 0.021, r = 0.81 (Effect size, large)) in the prefrontal area during the intervention period compared with the control period (Table 5). NIRS-SPM analysis indicated that channels 27 and 41 reflected activity in the left dorsolateral prefrontal cortex (DLPFC; Figs 5 and 6).

**Table 5:** Pre- and post-intervention differences in integral value (oxyHb) at channels in the frontal temporal region.

	Pre- intervention at baseline			Post- intervention			Difference pre-and post–intervention			
Channel	IVG period (n = 8)	CON period(n = 7)	Mann-Whitney -U (P-value)	IVG period (n = 8)	CON period(n = 7)	Mann-Whitney -U (P-value)	IVG period Wilcoxon signed-rank test(P-value)	CON periodWilcoxon signed-rank test(P-value)	Kruskal Wallis (P-value)	Effect size (r)
14	0.53(-1.04–2.92)	2.01(1.61–2.24)	0.203	-1.38(-5.75–9.46)	7.52(-1.89–13.86)	0.298	0.779	0.310	0.298	0.27
22	0.53(-1.10–2.70)	5.35(-0.73–5.73)	0.240	3.48(-1.86–6.49)	4.11(1.39–12.54)	0.487	0.484	0.612	1.000	0.00
24	3.78(-0.40–6.83)	3.62(1.62–7.95)	0.922	4.26(-0.29–9.39)	1.49(-15.59–8.88)	0.563	0.575	0.499	0.165	0.36
25	3.12(0.67–4.44)	3.01(-3.78–4.73)	0.769	2.90(0.05–11.48)	2.07(-20.90–11.59)	0.418	0.575	0.398	0.563	0.15
27	2.03(-0.91–4.11)	4.00(-0.65–18.45)	0.378	3.40(-1.73–17.54)	0.90(-7.85–6.94)	0.418	0.327	0.128	0.021	0.60
28	0.30(-3.51–2.91)	2.26(-12.08–4.86)	0.625	3.27(-1.76–6.13)	3.95(2.17–9.63)	0.643	0.484	0.128	0.563	0.15
30	2.30(-1.59–3.74)	-4.05(-7.39–19.08)	0.378	2.14(-2.40–4.16)	-1.62(-16.61–21.43)	0.643	0.093	0.735	0.165	0.36
31	1.49(-0.48–7.49)	3.15(-15.70–4.21)	0.695	4.12(3.33–10.63	7.12(-31.83–14.96)	0.908	0.779	0.237	0.908	0.03
34	1.08(-0.58–4.15)	1.91(-5.70–4.50)	0.922	3.95(-2.87–7.16)	-1.83(-17.55–21.43)	0.817	0.263	1.000	1.000	0.00
35	0.22(-1.69–3.86)	4.94(-0.47–8.48)	0.171	3.36(-2.04–4.16)	11.67(-4.41–23.24)	0.355	0.327	0.398	0.728	0.09
36	1.41(2.09–2.96)	2.29(-774.00–5.78)	0.434	3.53(-6.16–9.99)	-5.84(-14.41–9.27)	0.487	0.674	0.237	0.247	0.30
37	1.51(0.03–4.76)	3.21(2.61–8.01)	0.434	2.63(1.22–9.28)	5.02(-15.42-	0.817	0.327	0.612	0.165	0.36
38	1.52(-3.24–3.44)	3.03(2.81–4.49)	0.922	6.18(-1.22–9.02)	-0.65(-15.00–6.76)	0.165	0.208	0.310	0.643	0.12
39	-0.08(-5.62–3.89)	5.05(-7.93–7.03)	0.922	2.94(-11.68–12.48)	-8.83(-29.18–0.57)	0.165	0.889	0.310	0.105	0.42
41	1.39(-0.34–4.91)	0.76(-2.52–5.60)	0.171	2.27(-3.97–5.81)	-6.98(-17.70–0.29)	0.015	0.484	0.091	0.021	0.81

Notes: Data are presented as the median (interquartile range). CON, control; IVG, interactive sports video game https://figshare.com/s/81fb5c2571b7b2f3cc6b

### Network changes before and after intervention in the bilateral prefrontal region

**Fig 9:**
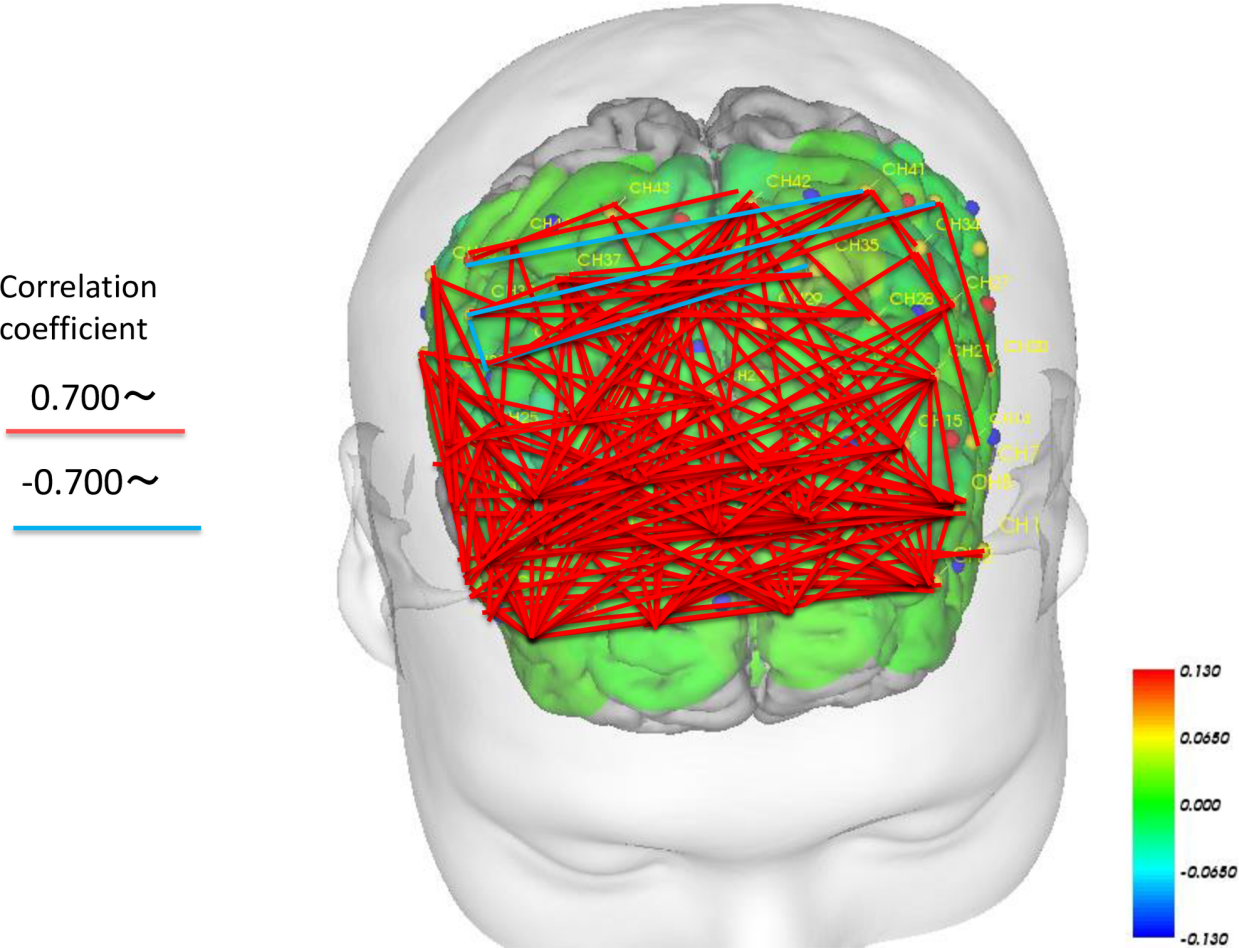
Correlation of cerebral blood flow changes in each channel showing a significant intervention-related change (before and after intervention) in the IVG period (Spearman’s correlation; p < 0.01).

**Fig 10:**
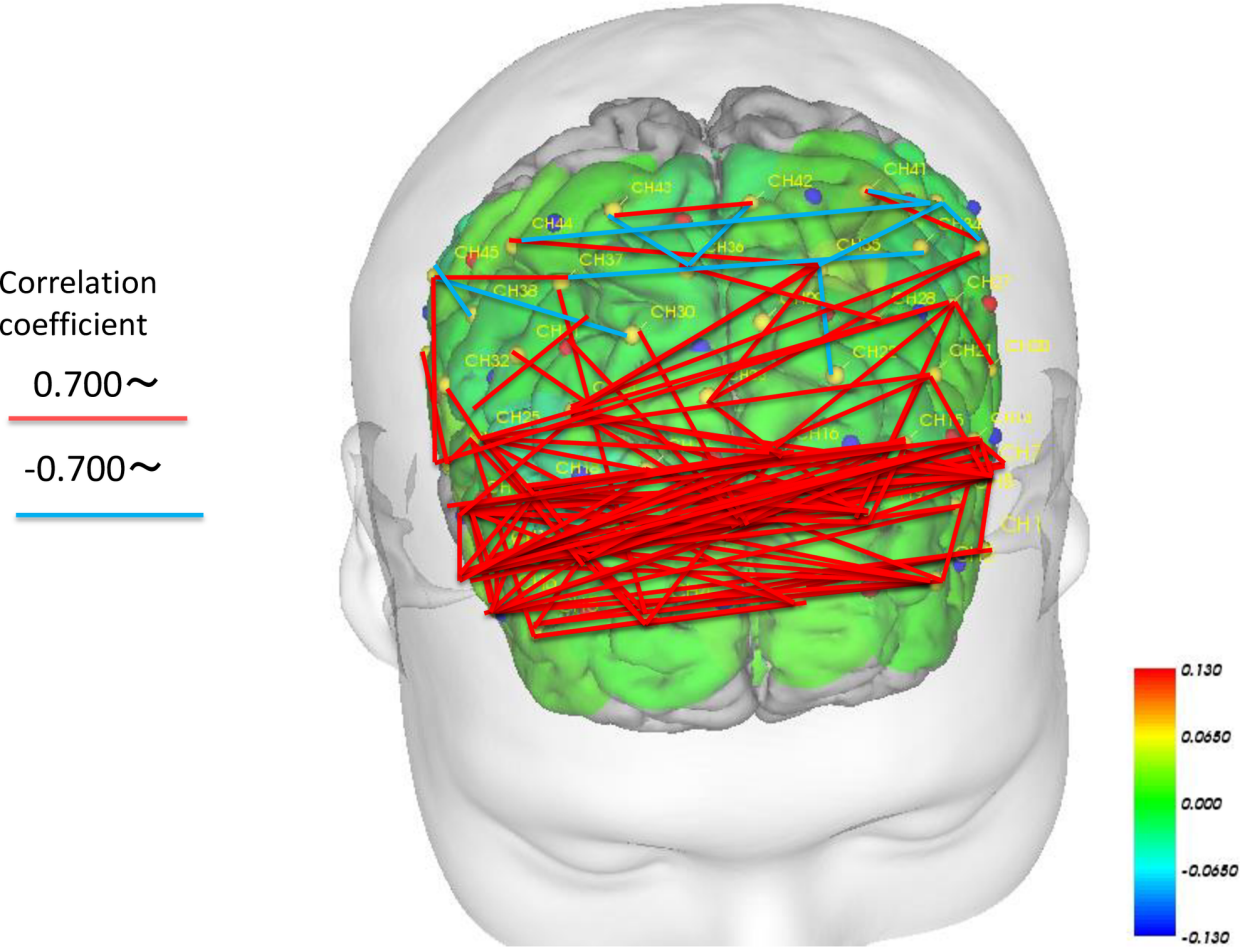
Correlation of cerebral blood-flow changes in each channel showing a significant intervention-related change (before and after intervention) in the CON period (Spearman’s correlation; p < 0.01).

We examined the correlations (Spearman’s; p < 0.01) between each channel to examine the magnitude of task-related activation changes before and after intervention in both the intervention and control periods. We drew lines between the channels in which significant differences were observed (Figs 9 and 10). The results revealed that the magnitude of change in cerebral blood flow before and after intervention changed in a similar manner while performing the task during the intervention period, suggesting similar increases in a wide range of areas, including the bilateral temporal areas, DLPFC and prefrontal areas (Fig 9). During the intervention period, one channel exhibited hub-like activity, with associations with many other neighboring channels (Fig 9). Meanwhile, the associations between channels during task performance were weaker during the control period compared with the intervention period (Fig 10). Although we observed associations in the bilateral ventrolateral prefrontal cortex (VLPFC) and the frontal orbital cortex, there were no associations between the bilateral dorsolateral prefrontal cortex and the medial prefrontal area. In contrast to the intervention period, we observed a negative correlation between these regions, revealing that as blood flow increased in one area, it decreased in the other (Fig 10).

## Discussion

### Behaviorally-assessed physical functioning before and after intervention

Our results did not reveal any significant intervention-related improvements in behaviorally-assessed physical functions. In particular, we predicted improvements in balance and lower limb muscle force/endurance, as subjects engaged in physical activity for 60 minutes while remaining in a standing position and moving their upper body left and right. Although comparison before and after the intervention revealed a significant improvement in balance, the comparison with the control period revealed no significant difference. This topic should be investigated in future studies with larger sample sizes.

### Comparison with HRQOL before and after intervention

We compared HRQOL (SF-36) performance before and after intervention in the intervention and control periods, which revealed a significant post-intervention improvement in 3 of 8 items: BP, SF, and RE. Impairment in each of these factors could inhibit activity, for both physical and psychological reasons.

Chronic schizophrenia is associated with durations of negative symptoms characterized by psychomotor sluggishness, decreased activity, numbed emotions, passivity, lack of proactivity, and lowered capacity for self-control and social interaction. The current results revealed greater improvements in BP, SF, and RE during the intervention period compared with the control period, indicating that the interest generated by engaging with the IVG led to improvements in motivation, daily activity levels, and alleviation of pain. These findings suggest that interventions using the Wii console for patients with schizophrenia could help to ameliorate the delayed social reintegration caused by decreased motivation and impaired activity resulting from negative symptoms.

### Frontal assessment battery (FAB) and changes in bilateral frontal lobe activity

The FAB measures cognitive functions associated with the frontal lobe. In this study, we did not observe a significant difference before and after intervention in either measurement period. Given that significant differences were observed in the IVG period before and after the intervention, it is likely that FAB differences would have been significant in a larger sample of subjects. However, recruiting enough individuals with schizophrenia to meet statistical requirements was difficult. Note that subjects reported subjectively positive experiences after completing the study, such as “I achieved a sense of accomplishment by concentrating on the game, and continuing [sic] to play it.” This suggests that gathering a larger participant sample might well statistically support such subjective reports.

We observed an increase in cerebral blood flow while subjects engaged in the IVG after 3 months of intervention, particularly in the DLPFC (channels 27 and 41). Abnormal DLPFC activity has been previously reported to be related to executive dysfunction, which is involved in specific challenges faced in daily life, such as preparing meals, washing, cleaning, organizing, book-keeping, as well as social challenges related to the use of public institutions or facilities, and the management of interpersonal relationships [30]. This brain region is also considered to be crucial for uniquely human cognitive function, including the inhibition of habitual or stereotypical repetition, liberating individuals from particular behavioral patterns to enable free, flexible, and varied responses. The current results suggest that an IVG intervention was effective for activating the DLPFC and increasing executive function performance and frontal lobe activity. Based on these results, we propose that this approach may provide an effective method for increasing the ability to adapt to community living among patients with schizophrenia who experience difficulties in daily life.

Kühn et al. [14] examined the effects of a 2-month period of video game training of at least 30 min per day. The results revealed that the gray matter volume (GM) in the right hippocampus (HC), right DLPFC, and bilateral cerebellum was significantly increased in subjects who underwent video game training, compared with controls. Furthermore, subjects’ motivation to play the video games correlated with the increase in GM in the HC and DLPFC, suggesting that game motivation might function as a useful predictor of the volume of GM increase.

The intervention tested in the present study consisted of a 60-min session each week for 3 months. Thus, the number of interventions was fewer than that used by Kühn and colleagues. However, we speculate that subjects’ continued interest and participation in playing the IVG for three months resulted in activation of the left DLPFC. In addition, subjects were instructed to freely select the games they preferred from the 12 options, to maintain motivation to participate in the intervention. Consequently, no subjects were observed playing the same game repetitively. Instead, they played three to four games in each session. This appeared to be an important factor contributing to the maintenance of interest in participation in the present study.

The task we used for cerebral blood flow measurement was a game called “Chanbara” in the Wii™ Sports Resort program. In the Chanbara game, objects such as watermelons, alarm clocks, and furniture are thrown towards the player, who is instructed to slash them faster than their opponent, following visual prompts indicated by arrows. Thus, performance of the task requires rapid judgment when deciding how to cut an object that suddenly appears in front of the eyes, and rapid movement when cutting the object. The fNIRS results indicated that regular performance of this task was effective in increasing brain activity during task performance in the intervention period compared with the control period.

### Role of the bilateral prefrontal cortical network in intervention-related changes

We analyzed the correlations between each fNIRS channel during task-related activity changes before and after intervention during both periods. The results revealed positively correlated cerebral blood-flow changes in many channels during the intervention period. This result may have been caused by the continuous repetitive stimuli involved in the IVG facilitating the activation of cerebral networks. In a meta-analysis, Minzenberg et al. reported that dysfunction in a number of regions related to executive function near the PFC, including the VLPFC, DLPFC, ventromedial PFC, and anterior cingulate cortex, is often observed in patients with schizophrenia [31]. In addition, during tasks that involve executive function, the same neural networks have been found to be activated in both healthy individuals and patients with schizophrenia [31]. However, it has also been reported that patients with schizophrenia commonly exhibit impaired activity in these networks, and some studies have reported increased activation of other PFC regions to compensate [31]. These previous findings are related to the results of the present study, which indicated that more neural networks were activated during the intervention period compared with the control period. We speculate that subjects may have responded to task performance by activating cortical regions outside of the dysfunctional networks. It is also possible that regular stimulation with the Wii console increased activation of networks other than the neural networks that are typically dysfunctional in schizophrenia, as a compensatory mechanism.

### Study limitations

Sample size was a key limitation of this study. From the power calculations (as described in **Materials and Methods: Subjects**), we required a within-group n = 19 for the Wilcoxon signed-rank test, and a between-groups n = 70 for the Mann-Whitney U tests. Initially, to reach these goals, we planned to collect data from 10 persons per facility (40 persons in total, and together with the controls, 80 people). However, patients with chronic schizophrenia were frequently uninterested in participating, so the number of people who consented to participate was only 3 per facility. Furthermore, the target was drastically reduced for this study because one facility opted not to participate. Because of the small final sample size, we cannot rule out a potential Type I error. Future studies incorporating a group size that can provide sufficient statistical power and additional tests are necessary. Such studies will likely need to recruit and test the subjects over a longer time period to ensure the reliability and validity of the research.

## Conclusions

The current study tested an intervention program designed to assist social reintegration of subjects with schizophrenia who were attending psychiatric care programs. The same subjects engaged in one 60-min session of Wii™ Sports Resort per week for 3 months, and behavioral assessments of physical functioning, HRQOL, cognitive functioning, and cerebral blood flow were measured. Statistical comparisons between the intervention and control periods revealed three key findings. First, there were improvements in three HRQOL items (BP, SF, and RE) following intervention. Second, we found an intervention-related increase in DLPFC activation. Third, there was increased activation in cerebral neural networks related to dysfunction in schizophrenia.

In future studies, we intend to examine a larger sample size and investigate the effects of regular intervention with highly versatile IVGs designed to promote social reintegration of patients with schizophrenia who experience difficulties in daily life owing to dysfunctional executive functioning. The current results provide promising evidence for the utility of IVG-based interventions in schizophrenia, thus warranting further investigation.

## Supporting information

S1 File This is the S1 File CONSORT checklist. (DOC)

S2 File This is the S2 File protocol. (DOC)
