# Supplementary material for: An interactive sports video game as an intervention for rehabilitation of community-living patients with schizophrenia: A controlled, single-blind, crossover study
Source: PLoS One. 2017 Nov 13;12(11):e0187480. doi: 10.1371/journal.pone.0187480 (PMC5683619; doi:10.1371/journal.pone.0187480)
Supplement: S2 File — (DOC) [file pone.0187480.s002.doc]

| Study Protocol | | |
| --- | --- | --- |
| Title of the study | | Virtual Reality Sports Game as an Intervention for Rehabilitation of Community-Living Patients with Schizophrenia |
| The study purpose and significance | 【The study purpose and significance】In recent years, progress has been made in hospital-discharge support for patients with mental illnesses who have undergone long-term hospitalization. However, effective methods of rehabilitation for chronic mental illness are severely lacking. Patients with mental illness commonly experience social exclusion and difficulty in their daily lives as a result of their condition, often leading to repeated hospitalization. A 2013 survey by the Ministry of Internal Affairs and Communications of Japan reported that many people with mental disorders have little to no contact with family or their communities, becoming socially isolated as a consequence(Ministry of Internal Affairs and Communications.2013). Patients with mental illness who have difficulty in social functioning typically face difficulty managing specific daily tasks such as preparing meals, washing, cleaning, organizing, and bookkeeping, and in social interactions such as using public institutions and maintaining relationships. Impaired executive function has a particularly strong impact on activities of daily living. Executive function is defined as the human ability to skillfully engage in purposeful, self-serving behavior (Lezak, M.D 1982). This ability involves the frontal lobes, and is characterized as the highest-level cerebral function. Specifically, executive function includes planning and problem-solving abilities, such as making predictions, selecting goals, planning, organization, and beginning, executing and integrating goal-oriented behaviors. Frontal lobe activation underlies these executive functions, and healthy frontal-lobe activity is thought to be a contributing factor in the prevention of social isolation in patients with mental disorders(Lezak, M.D 1982). Moreover, there is evidence that patients with chronic schizophrenia commonly exhibit hypofrontality, which is characterized by reduced blood flow into the frontal lobes relative to other areas of the brain at rest. Consequently, hypofrontality has received attention in schizophrenia research (Ingvar DH 1974). However, there are few effective methods for improving cognitive function in individuals with schizophrenia. In addition, there have been few studies of interventions for improving motor function (Takahashi H,2008).Meanwhile, there have been many reports of the development of programs targeting older individuals, who tend to engage in limited habitual exercise. Dual-task training has been reported to be an effective intervention, not only for motor function, but also cognitive function. This training includes physical movement to match the rhythm of music (eurhythmics) and the performance of simple math calculations while walking. Bridenbaugh and Kressig proposed that neural circuits are first developed as a foundation for music learning by reproducing musical gestures and physical movements, and that repeated perception of movement elements of music through muscular movement stimulates these neural circuits. Thus, there is evidence that dual-task training stimulates motor and cognitive functions simultaneously, thereby improving functioning in both domains.However, although it is possible for patients with schizophrenia to participate in physical-exercise programs while hospitalized or attending psychiatric-care centers, it is difficult for such individuals to voluntarily participate in group-exercise programs after returning to their communities following discharge (Green MF 2000). As a result, most patients with schizophrenia stop exercising after discharge. | |
| The study purpose and significance | 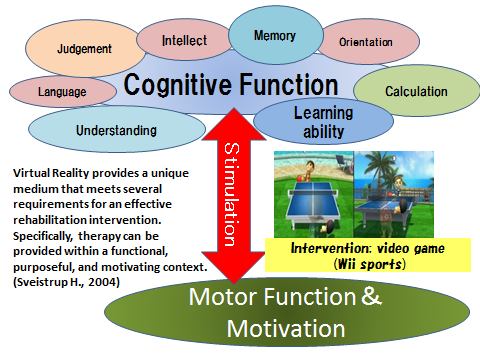Virtual reality games (VRG) can easily be used independently in the home. The current study tested the effects of a VRG exercise program on physical and cognitive function as well as health-related quality of life (HRQOL) in patients with schizophrenia, with a practice period of 3 months. This method was examined as a potential intervention for increasing daily motivation to improve and maintain cognitive function in patients with schizophrenia living in the community, thus potentially aiding their reintegration into society. | |
|  | **1) Study design　Cross-over, Quasi-randomized (a controlled-clinical-trail)Single blind -investigator(s) and assessor(s) are blinded2) Subjects Subjects were attendees of psychiatric care services at a location in Ishikawa prefecture and two locations in Fukui prefecture. They met all five of the following inclusion criteria: 1) diagnosed as “schizophrenic” according to the criteria defined by the International Classification of Diseases (the 10th revision of the International Statistical Classification of Diseases and Related Health Problems; ICD-10); 2) not diagnosed with a mental disability, alcoholism, or drug addiction; 3) aged 18–64 years; 4) scored 40 points or more on the Global Assessment of Functioning (GAF), indicating a non-severe mental state; and 5) capable of fully understanding the purpose and content of the research. The nature and purpose of the study was explained to all subjects, and written consent to participate in the study was obtained. Each patient’s principal doctor’s permission was also obtained. Based on a precedent study [17], we found the number of the samples. 3) Intervention Method The intervention used a Nintendo Wii™ console software program called “Wii™ Sports Resort” (Table 1). This game includes 12 sports. Performance requires fine movements with the Wii remote control, resulting in motions similar to those involved in performing the actual sport (Table 1). To perform the task, the subject was required to stand in front of the screen and use their whole body to manipulate the remote control, while playing against a computerized opponent on the monitor in each game. This intervention was implemented as one 60-minute session per week for 3 months. In the control period, the same intervention group participated in a normal occupational therapy program over the same period 1 year later (Jun–Sep), and measurements were taken in the 3 months before and 3 months after participation. Thus, the group that initially underwent the Wii intervention participated in the activities of the control group, and the group that was in the control period to start with participated in the Wii intervention during the same period in the following year.**Table 1. Twelve Sports Included in Wii™ Sports Resort.   | **Easy sports** | **Ball sports** | **Leisure sports** | | --- | --- | --- | | Chanbara | Basketball | Canoeing | | Biking | Ping pong | Wakeboarding | | Frisbee | Golf | Sky ranger | | Archery | Bowling | Marine biking |   4). Details of the survey 1.Basic characteristics family situation, chief complaint, medical history, drug therapy, family history, height, weight, and blood pressure.2.Measurement of general mental function 3.Cognitive function scale4.HRQOL Scale (SF-36) 5.Measurement of physical function This is evaluated on the following six items of skillfulness and agility (Timed Up and Go), flexibility (functional reach test), balance (one-leg standing test with eyes open), gait (walk 10m at maximum force),muscle endurance (30-second chair test) and muscle force (grip strength). 6. Cerebral blood flow measurement We measured cerebral blood flow while playing virtual reality games using functional near-infrared spectroscopy(fNIRS).5)Analysis (1) Changes before and after interventionWe compared the physical function measurements, SF-36, and FAB score between the intervention period and control period. We conducted a paired t-test to compare values before and after intervention. We then performed a one-way analysis of variance to examine the values before and after intervention and evaluate task-related changes. (2) Evaluation of cerebral blood flow measurementTo evaluate task-related changes, we analyzed the change in integral oxyHb integral before and after task performance in the intervention and control periods based on brain coordinate information, using analysis of variance (ANOVA) with Bonferroni correction for multiple comparisons.6). Ethical considerationA researcher will explain the study purpose, method. And we explain the following contents, and only obtained person consent study. All participants signed an informed consent before participating in the study.1. Confidentiality: We will make every effort to maintain the privacy of your data. We will maintain the privacy of the data collected, monitor data entry, and conduct analyses. Only the researchers will see the data. No release of data collected from the project will identify individual participants in the study. Information on individual responses will not be shared with anyone or affect your grade for any class.2.Invitation for Questions: If you have questions about this study, you should contact the Researcher, before you sign this consent form. If you have questions regarding your rights as a participant, any concerns regarding this project or any dissatisfaction with any aspect of this study, you may report them – confidentially, if you wish – to the Project Supervisor,.3.Study Withdrawal: You have the right to withdraw your consent or stop participating at any time. You have the right to refuse to answer any questions or participate in any procedure for any reason. Before starting the survey, we explained the nature and purpose of this study as well as any risks associated with it. The survey and research were conducted after subjects had provided informed consent. 7)study schedule[from 2014 to 2016]・2014June: I apply to the Ethical Review Board in the study (Shimizu)July: Explanation (Shimizu, Hori, Shimokawa) July to November: for study entry agreements to the target personIntervention start (Shimizu, Hirai,　Shimokawa, Ohe) November to March:Data collection, analysis (Shimizu, Shimokawa, Hori, Umemura) ・2015June to September: Cross over study intervention to the target person(Shimizu, Hirai, Shimokawa, Ohe) September to March:Data collection, analysis (Shimizu, Shimokawa, Hori, Umemura) ・2016　April : Presentation at the meeting, article making, contribution preparations (Shimizu, Shimokawa, Hirai, Umemura) October: Briefing session (Shimizu, Ohe) in the study8) Role in research Study representative (Shimizu): Study generalizationCoworker: Planning and implementation (Ohe, Hori, Shimokawa, Igarashi, Matsuyama, Sakakibara): The medical management of the target person.Coworker (Hirai, Umemura): Operation about fNIRS, Data analysis.  The Shimadzu LABNIRS (Shimadzu Co. Ltd., Kyoto, Japan) used in the current study was developed for research purposes, and measures three wavelengths (780 nm, 805 nm, and 830 nm) to detect changes in light absorption, allowing calculation of changes in concentrations of oxygenated and deoxygenated hemoglobin (oxyHb and deoxyHb). | |
